# Quantum Smell: Tunneling Mechanisms in Olfaction

**DOI:** 10.3390/molecules30244663

**Published:** 2025-12-05

**Authors:** Dominik Szczȩśniak, Ewa A. Drzazga-Szczȩśniak, Adam Z. Kaczmarek, Sabre Kais

**Affiliations:** 1 Institute of Physics, Faculty of Science and Technology, Jan Długosz University in Czȩstochowa, 13/15 Armii Krajowej Ave., 42200 Czȩstochowa, Poland; adam.kaczmarek@doktorant.ujd.edu.pl; 2 Department of Physics, Faculty of Production Engineering and Materials Technology, Czȩstochowa University of Technology, 19 Armii Krajowej Ave., 42200 Czȩstochowa, Poland; ewa.drzazga@pcz.pl; 3 Department of Electrical and Computer Engineering, North Carolina State University, 890 Oval Dr., Raliegh, NC 27606, USA; skais@ncsu.edu; 4 Department of Chemistry, North Carolina State University, 2620 Yarbrough Dr., Raleigh, NC 27695, USA

**Keywords:** olfaction, electron tunneling, charge transport, vibronic theory, reorganization energy, electron-phonon coupling, swipe card model

## Abstract

The mechanism by which odorants are recognized by olfactory receptors remains primarily unresolved. While charge transport is believed to play a significant role, its precise nature is still unclear. Here, we present a novel perspective by exploring the interplay between the intrinsic energy scales of odorant molecules and the gap states that facilitate intermolecular charge transport. We find that odorants act as weak tunneling conductors mainly because of the limited magnitude of electronic coupling between frontier molecular levels. This behavior is further connected to electron–phonon interaction and reorganization energy, suggesting that physically meaningful values for the latter parameter emerge only in the deep off-resonant tunneling regime. These findings complement the swipe card model of olfaction, in which an odorant needs both the right shape to bind to a receptor and the correct vibrational frequency to trigger signal transduction. Moreover, they reveal that the underlying mechanisms are much more complex than previously assumed.

## 1. Introduction

As far as human perception is concerned, the sense of smell, or olfaction, is one of the first concepts that comes to our minds. However, the obviousness of its existence does not go hand in hand with a complete understanding of why we are able to perceive a plethora of various scents. Although it is already well known that it takes activation of the odorant receptors to stimulate neurons in our noses [1], the underlying mechanism of olfactory discrimination is somewhat unknown. So far, several factors have been identified as potentially pivotal for our smell impressions, namely the physicochemical attributes (size, shape, bonding, etc.) [2,3,4] and vibrations [5,6,7] of the odorant molecules. The debate on which of these features is dominant, or even relevant, in the olfactory process appears to be at the cornerstone of the future theory of smell [8,9]. Unfortunately, none of them seems to be sufficiently explored, including their likely interdependencies.

All the above intricacies of smell naturally lead to an essential question: can we arrive at a unified picture of the olfaction mechanism that allows for simultaneous experimental verification? Herein, it is argued that an important insight in this respect can be obtained by recalling the general interpretation of charge transport at the quantum level. As may already be apparent, such reasoning shares some similarities with the vibrational theory of smell [7]. Still, it does not pre-assume the validity of this idea, being rather aimed at assessing the general plausibility and role of electron transport in shaping olfaction. In fact, this process is cardinal to the neural system and, as such, naturally calls for inclusion in the analysis of the olfactory segments. This is to say, even if electron transport is unable to solely explain the olfaction mechanism, it can still be expected to be of fundamental importance to this problem.

In detail, when considering charge transport in the quantum regime, we intuitively reflect on the phenomenon of electrons scattering from a potential barrier. This effect lays the foundations for our understanding of electronic transport in mesoscopic systems, as formulated by Landauer [10]. While the aforementioned approach originally relies on electrons being propagated in the semi-infinite terminals before reaching a scatterer, it seems to be viable for fully molecular considerations as well. By following in spirit the studies of Nitzan et al. [11,12,13,14], it can be deduced that the electronic transport across a molecule coupled to a periodic medium mimics, to a great extent, the tunneling process in the donor–molecule–acceptor system. Hence, the schematic representation of an odorant docked in the olfactory receptor. This point of view is reinforced by the fact that in the zero bias limit (i.e., in the regime where Nitzan’s argumentation appears to be particularly valid [13]), electron tunneling is largely independent of the considered terminals [15]. To be specific, this is the non-resonant regime where tunneling conductance rate (τ) is

(1) $$\tau \propto e^{-2\beta L},$$

with β being the decay rate and *L* denoting the scatterer length. Although the above derives from partially periodic considerations, it is in qualitative agreement with what can be found for the electron exchange rates between purely molecular species [16,17,18].

## 2. Results and Discussion

**Figure 1:**
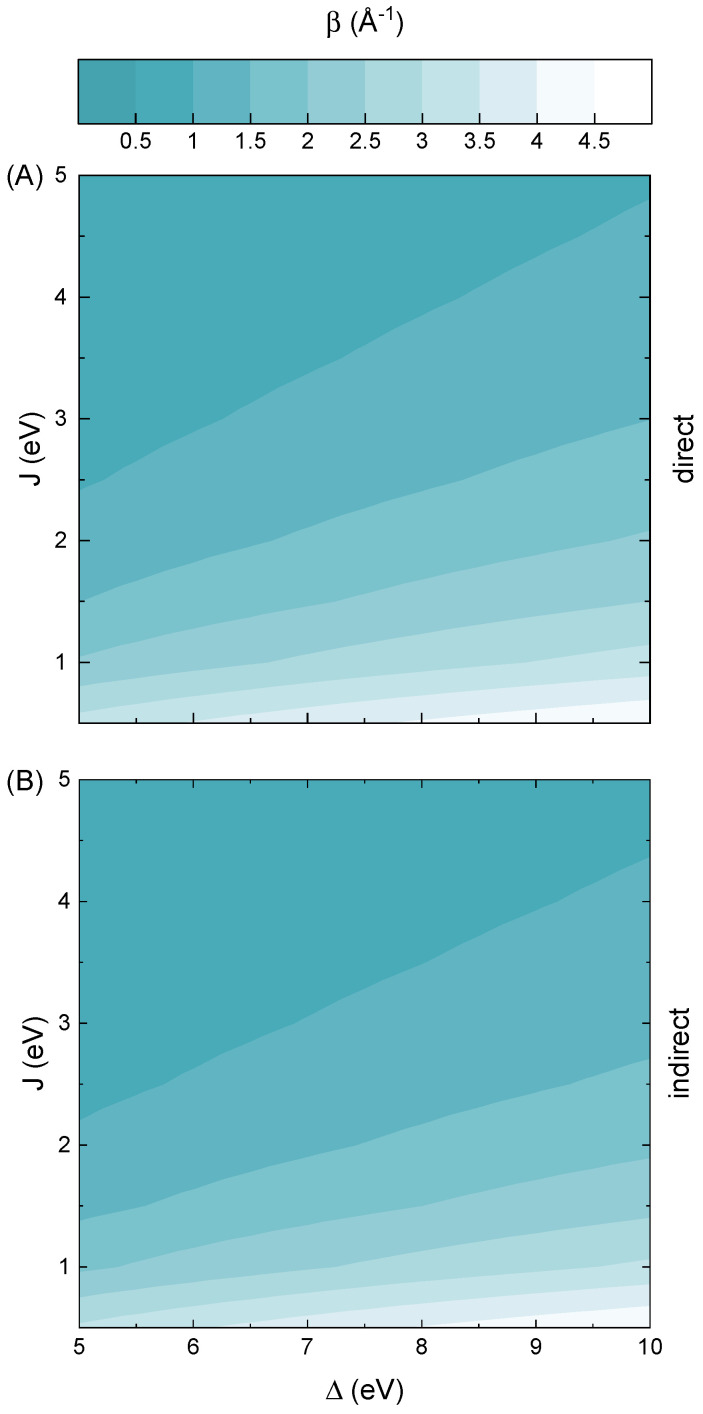
The decay rates for odorant molecule as a function of electronic coupling and energy gap size. Two tunneling regimes are considered, the direct (**A**) and indirect (**B**) one.

In Figure 1, the magnitude of the decay rates for the direct and indirect tunneling processes is depicted as a function of the energy gap and electronic coupling. The contour plots are derived from Equation (2) by employing technique of analytic continuation into the complex-energy domain, as described in Section 3. The characteristic scales of energy gap (Δ) and electronic coupling (*J*) are specifically chosen to span the electronic characteristics typical of odorant molecules, with the latter additionally extended toward higher values to provide a broader context. In detail, the lower gap boundary corresponds to the average electronic gap of conjugated or partially conjugated odorants, such as those found among body odor volatilomes [19]. The intermediate region represents systems of mixed polarity, exemplified by molecules with *green* scents [20]. Finally, the upper end of the assumed range corresponds to saturated and weakly polar odorants exhibiting large gaps of about 10 eV or more [21]. In what follows, this range covers common odorants such as vanillin (∼5 eV) [22], menthol (∼7 eV) [23] or hydrogen sulfide (∼8 eV) [24]. On the other hand, the adopted range of electronic coupling values spans from weakly interacting and localized orbitals to strongly hybridized frontier states, according to Equation (2). In this framework, the energy of the highest occupied molecular orbital (HOMO) level is set to zero and used as a reference value. The direct tunneling rates correspond to the branch point of the gap states, whereas the indirect process is evaluated at an energy equal to $\varepsilon_{L}/6$, i.e., close to the HOMO level. Moreover, the indirect tunneling rates are sampled only at energies below this point, since the HOMO and lowest unoccupied molecular orbital (LUMO) levels are equidistant from the branch point.

A key observation from the results presented in Figure 1 is that, within the assumed range of Hamiltonian parameters (see Equation (2)), the decay rates take values from 0.5 Å^−1^ or less up to nearly 5 Å^−1^. This wide variation indicates that the character of charge transport transitions from being weakly dependent on distance to exhibiting exponential decay with strong distance dependence. Such a shift has a significant impact on the underlying transport regime, which is indicative of a near-resonant process when decay rates are low ($\beta <0.5\;{Å}^{-1}$) and suggests coherent and fully off-resonant tunneling for higher decay values [25]. Interestingly, it can also be observed that decay rates exhibit relatively little sensitivity to changes in the energy gap under weak coupling conditions and yield low values only for strong electronic coupling. This is somewhat expected, since higher electronic coupling should promote more efficient charge transport, as suggested by the stronger overlap of orbitals. However, this behavior stands in partial contrast to the preliminary argument by Brookes, which suggests that tunneling across an odorant molecule would be ineffective mainly due to the large energy gap characteristic of such molecules [26]. In fact, the calculated decay rates at the midpoint value of electronic coupling become relatively efficient for transport to occur and only improve as the coupling increases. Moreover, these findings are mostly uniform, whether the direct or indirect process is considered. Only some small improvements in decay rate values are present in terms of the indirect tunneling, but rather in the strong coupling limit.

In what follows, to estimate the regime of charge transport across odorants, it is essential to consider the interplay between the band gap and electronic coupling. Specifically, for tunneling to be meaningfully described by the two-level Hamiltonian of Equation (2), the HOMO and LUMO states must remain distinct rather than strongly mixed. This occurs only when the electronic coupling is much smaller than the band gap [26,27,28]. For odorants, Equation (2) indicates that this weak-mixing condition is well satisfied for J<1 eV, since in this regime J≪Δ and the mixing angle tan(2θ)=2J/Δ remains small, preserving the character of the two states [29]. This picture is consistent with the standard two-level framework for discussing tunneling across odorants [30,31].

Under such conditions, the results presented in Figure 1 clearly suggest that charge transport via odorants likely falls into the deep off-resonant tunneling regime. In detail, assuming an average odorant molecular size of L∼10 Å [32], one finds that the decay rates reported in Figure 1 yield physically meaningful tunneling conductance (down to $\tau ∼10^{-8}$) only within the moderate-to-strong coupling regime (J>2 eV). This threshold for τ reflects practical detection limits in single-molecule transport experiments, including noise floors and instrumental sensitivity in molecular junction measurements [33]. Although tunneling may remain detectable under more favorable conditions or via additional mechanisms, even for a shorter effective length of L∼5 Å the transmission factor already drops to $\tau ∼10^{-9}$ for couplings J∼1 eV, i.e., enters regime in which coherent tunneling is generally considered negligible for most molecular systems. In other words, the simple exponential relation for the tunneling breaks, the corresponding transmission becomes unmeasurably small, and other mechanisms (like hopping or phonon-assisted tunneling) may dominate. However, this also indicates that odorant molecules are poor tunneling conductors not only due to the large energy gaps but also to small electronic coupling. In fact, the presented results suggest that the electronic coupling plays a more significant role in suppressing tunneling rates in odorants than the energy gaps themselves. Altogether, this implies that the extramolecular mechanism proposed by Brookes [26] remains viable, although the underlying rationale appears to be more complex than previously anticipated.

In this context, it is essential to discuss the relation between tunneling and the vibrations of the odorant molecule. In the framework of Equation (2), the odorant can be regarded as a two-level vibronic system, or oscillating dimer, in which the HOMO and LUMO states are linked by a phonon-modulated tunneling amplitude. In this first-order approximation, the effect of the vibrational mode is distilled into a single parameter of the electron–phonon interaction strength, as derived from the electronic coupling. Specifically, in the frozen-phonon regime, the linear electron–phonon coupling can be written as α=dJ/du, with *u* standing for the displacement per atom. To introduce the necessary dependence of electronic coupling on the distance, this parameter can be assumed to have the character of a two-central Slater–Koster integral that decays exponentially with the distance as $J\left(l\right)=J_{0}e^{-\eta (u/a_{0}-1)}$, where $J_{0}$$\left(a_{0}\right)$ is the equilibrium coupling (distance) between two sites and η denotes the fall-off rate, which can be interpreted as a phonon mode softening/hardening rate [34,35]. Note that the exponential form is one of the many available ones, but it is chosen here since it was proved to be well-suited for the π-conjugated systems [35,36], non-trivially capturing the quantum-mechanical decay of orbital overlap with distance, which should be especially relevant in small molecules where electronic interactions are highly sensitive to bond-length fluctuations.

It is important to note that the linear electron–phonon coupling introduced here is strongly model dependent and, by itself, cannot be used for a robust quantitative comparison with literature values. However, it can be related to the somewhat sibling reorganization energy [37,38,39], familiar in the *swipe card* model of olfaction [26,27] and rooted in the Marcus theory [16]. For this purpose, it is convenient to recall the potential energy function for the quantum oscillator $V\left(u\right)=1/2M\omega^{2}u^{2}$, where *M* is the effective mass and ω stands for the characteristic frequency. Assuming that the final electronic state of the odorant shifts the potential minimum due to the linear electron–phonon coupling term, the reorganization term can be simply given as $\lambda =\alpha^{2}/2M\omega^{2}$. Here, it is assumed after Brookes [27] that M=8.25 amu and ℏω=0.2 eV. These can be considered representative values of the odorant molecules that also allow comparison with other estimates available in the literature. In this manner, the direct relation between the electronic coupling and two scales related to the vibrations of the odorant is established.

**Figure 2:**
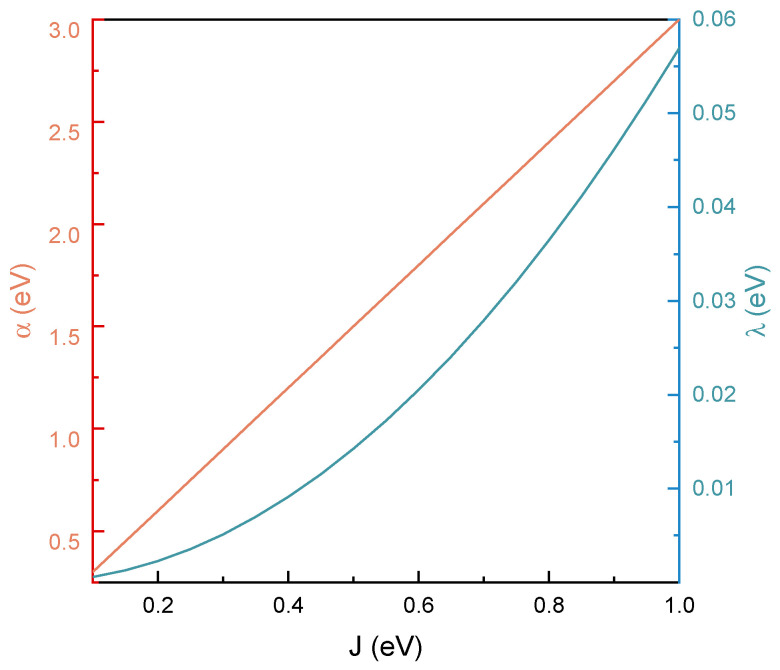
The magnitude of the linear electron–phonon interaction (orange) and reorganization energy (turquoise) as a function of the electronic coupling (black).

In Figure 2, the behavior of the linear electron–phonon interaction and reorganization energy as a function of the electronic coupling is presented. Note that only J<1 eV is considered, in agreement with the earlier condition for strongly localized HOMO and LUMO. In general, both discussed terms are clearly increasing along with the increase in the electronic coupling, although they do so in qualitatively distinct ways. The electron–phonon coupling exhibits linear strengthening, which is in line with the earlier observations made for carbon systems [35,36] and reflects the exponential dependence of orbital overlap on interatomic distance. More precisely, since $J\left(u\right)\propto e^{-\eta u}$, its gradient with respect to displacement naturally scales with its own magnitude, yielding α∝J. This implies that stronger electronic tunneling leads directly to a proportionally enhanced coupling to nuclear motion. On the other hand, the reorganization energy shows a nonlinear (quadratic) increase and indicates that strong tunneling not only enhances the coupling to vibrations but also amplifies the structural response, making the system increasingly vibronically active. However, of particular importance to the presented discussion are the quantitative, not qualitative, features of the presented results. Specifically, the reorganization energies for organic molecules are known to exhibit values of a few tenths of an electronvolt [40,41]. However, for odorants, one expects even lower estimates, since olfactory receptors reside in a hydrophobic and rigid protein environment [27]. In such settings, nuclear rearrangement is strongly suppressed, and reorganization energies can fall well below solvent-based values [42]. For example, *rhodobacter capsulatus* is characterized by reorganization energy around 0.03 eV [43], whereas similarly low values have been reported for other related systems, including estimates of λ≈0.07 eV for the protein part of *ferredoxin* [44] or *rhodobacter* [45]. These observations are in agreement with the discussion provided for odorant molecules by Brookes [27]. In what follows, the suggestion that tunneling across odorant molecules likely falls into the deep off-resonant limit is reinforced, supporting the argumentation behind the *swipe card* model of olfaction.

## 3. Methodology

The rationale presented in Section 1 means that electron tunneling in the odorant–receptor system may be conveniently captured within the Landauer approach. According to the mentioned decay characteristics of this process, of pivotal importance to such discussion seem to be the exponent observables. The scatterer length, being the first of them, can be simply interpreted as the distance between the acceptor and donor sites in the olfactory receptor. This parameter is naturally limited by the ability of electrons to traverse the region between the receptor terminals in the tunneling regime. The second feature is much more complex, as it captures the intrinsic properties of the bridging structure such as its chemical structure and energetics. Still, contrary to the available semi-classical [16,27] or even quantum-mechanical [46,47,48,49] studies of the olfaction problem, the provided reasoning appears to have a more canonical character. This is not only due to the fact that it stems from the fundamental depiction of electron transport at the quantum level but also attempts to cut out secondary aspects such as the structure of acceptor and donor sites.

In this context, it is suggested that one of the two primary scenarios may occur, namely intra- or extramolecular transport, both of which were initially considered by Brookes [16,26,27]. Out of these two, the second approach was quickly disregarded due to the large energy gap of odorants, without any further analysis conducted [26]. However, sizable band gaps in molecular systems do not necessarily lead to negligible tunneling rates, as has been shown, e.g., for alkanes [50]. On the other hand, the analysis of tunneling processes across DNA sequences suggests that, although their band gaps are not exceptionally large, the corresponding decay rates are high, leading to poor charge transport [51]. In what follows, it is argued here that the charge transport across odorant molecules should not be discussed solely based on the energy gap scale, without taking into account other important factors. Of particular importance is the coupling between electronic states and their interaction with molecular vibrations, which cannot be ruled out. Specifically, the former facilitates tunneling and defines the strength of the corresponding transport channel, while the latter can be expected to further assist in mediating charge transport between donor and acceptor sites. That is to say, the vibrations of the odorant molecule do not merely have to bridge the energy gap, but can instead couple to the electrons responsible for shaping tunneling across the associated potential barrier. Notably, this insight also constitutes a viable platform for experimental verification of the tunneling mechanism due to the similarities between purely molecular systems and those terminated by metal electrodes, as discussed by Nitzan [11,12,13,14,52].

**Figure 3:**
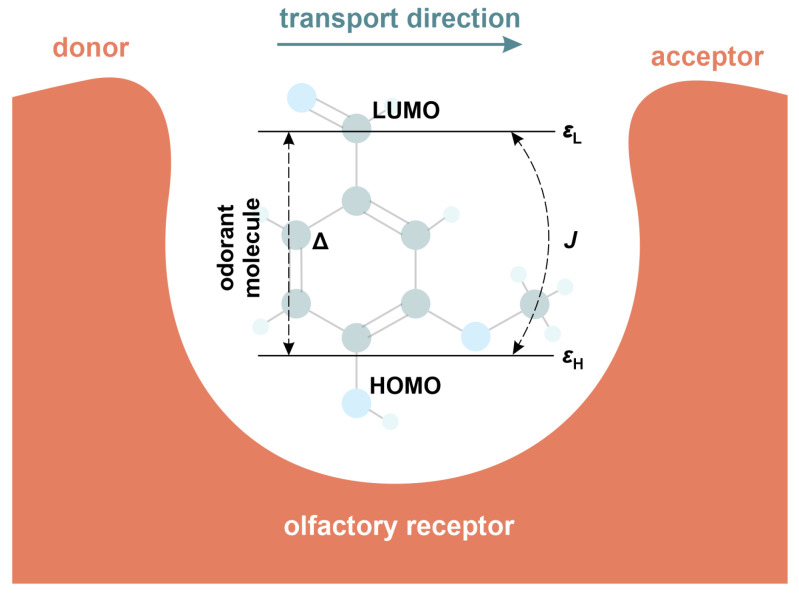
Schematic representation of an odorant molecule locked in a olfactory receptor. The former is presented here as a two-level molecule defined by the highest-occupied (HOMO) and lowest-unoccupied (LUMO) molecular orbitals with energies $\varepsilon_{H}$ and $\varepsilon_{L}$, respectively.

The above can be pictured by considering interplay between mentioned energy scales. These are conveniently captured by the following Hamiltonian which yields the highest-occupied (HOMO) and lowest-unoccupied (LUMO) molecular orbitals of an odorant:

(2) $$H=\varepsilon_{H}c_{H}^{†}c_{H}+\varepsilon_{L}c_{L}^{†}c_{L}+J\left(c_{H}^{†}c_{L}+c_{L}^{†}c_{H}\right),$$

where $c_{H/L}$$\left(c_{H/L}^{†}\right)$ creates (annihilates) electron with energy $\varepsilon_{H/L}$ at the HOMO/LUMO level and *J* denotes electronic coupling between considered energy levels. In this framework, the energy gap is simply given as $\Delta =\epsilon_{L}-\epsilon_{H}$. All these quantities are referred to here as the intrinsic energy scales of the odorant. As a result, they represent exclusively molecular properties of the odorant itself, with no receptor electronic states included. This choice is consistent with previous quantum-mechanical models of olfaction, in which the internal energy scales of odorants are treated as among the dominant factors governing tunneling and inelastic excitation pathways [26,30,49]. The corresponding schematic representation of the odorant molecule is presented in Figure 3.

With the above in mind, the tunneling across an odorant molecule may be viewed as an evanescent wave coupling effect [53,54]. As such, the evanescent modes mimic channels that enable the single-step transport of electrons across the molecule. Importantly, these can be effectively probed in the experiment by using, e.g., the scanning tunneling microscopy [55]. In practice, there are several ways to determine such evanescent modes, one of which is the analytic continuation of the underlying eigenvalue problem into the complex domain [50,53,56]. Within this framework, the finite molecular Hamiltonian of Equation (2) can be recast into a transfer matrix form (*T*) as

(3) $$\left(\begin{array}{c} \psi_{H}\\ \psi_{L} \end{array}\right)=T\left(\begin{array}{c} \psi_{L}\\ \psi_{H} \end{array}\right),$$

where $\psi_{H}$ and $\psi_{L}$ are the expansion coefficients of the molecular state vector (|Ψ〉) expressed in the two-level basis of the model Hamiltonian, according to

(4) $$|\Psi \rangle =\psi_{H}\;|H\rangle +\psi_{L}\;|L\rangle ,$$

with |H〉 and |L〉 denoting abstract basis states that correspond to the HOMO and LUMO, respectively. These states represent frontier levels within a tight-binding-like framework, while $\psi_{H}$ and $\psi_{L}$ are the associated amplitudes rather than spatial orbitals obtained from density functional theory or Hartree–Fock methods. Consequently, all results follow analytically from Equation (2) without requiring any explicit quantum-chemical basis set.

As such, Equation (3) yields solutions (eigenvalues) of the form $e^{\pm \beta }$, which correspond to evanescent modes when not equal unity. These modes are interpreted as gap states that appear within the forbidden energy region of the molecule, representing yet another of its intrinsic electronic features. Central to the present analysis is the branch point of the gap states, defined by the condition dβ/dE=0. At this point, the molecule remains charge neutral, giving rise to the most penetrating evanescent modes characterized by the minimum decay rates. According to Equation (1), this defines the dominant direct tunneling channel, since the transmission is exponentially larger for smaller β, in contrast to the indirect processes occurring at other energies within the gap [57]. In other words, the effective energy barrier height (Φ) is the smallest, knowing that [50,57,58,59]

(5) $$\Phi =E+\frac{{\left(\hbar \beta \right)}^{2}}{2m},$$

where *m* is the electron mass and *E* is the tunneling energy.

## 4. Summary and Conclusions

In summary, the general analysis presented here offers novel insights into the regime of quantum tunneling across odorant molecules. It establishes fundamental limits for such processes based on the intrinsic energy scales that govern these molecular systems, thereby shedding light on the mechanism of olfaction. In particular, in the context of the *swipe card* model of olfaction, presented results appear consistent with this framework. Specifically, they provide partial support for the idea that quantum tunneling may contribute to the sense of smell. While tunneling alone may not fully explain olfactory recognition, it should be considered as one of several contributing factors. To further investigate this direction, the study provides useful theoretical relations and identifies measurable observables that could be tested in future experiments. Moreover, the same framework may serve as a foundation for large-scale screening of odorant–receptor interactions using realistic molecular structures and advanced computational techniques. In particular, machine learning approaches might be valuable in validating and expanding upon the arguments presented in this study.

## Data Availability

The datasets analyzed during the current study are available from the corresponding author on reasonable request.
